# Nephron development and extrarenal features in a child with congenital nephrotic syndrome caused by null *LAMB2* mutations

**DOI:** 10.1186/s12882-017-0632-4

**Published:** 2017-07-06

**Authors:** Jiro Kino, Hiroyasu Tsukaguchi, Takahisa Kimata, Huan Thanh Nguyen, Yorika Nakano, Noriko Miyake, Naomichi Matsumoto, Kazunari Kaneko

**Affiliations:** 1 Department of Pediatrics, Kansai Medical University, 2-5-1 Shimachi, Hirakata, Osaka, 573-1010 Japan; 2 Second Department of Internal Medicine, Kansai Medical University, 2-5-1 Shinmachi Hirakata, Osaka, 573-1010 Japan; 3 Department of Pathology and Laboratory Medicine, Kansai Medical University, Osaka, Japan; 4 Department of Human Genetics, Yokohama City University Graduate School of Medicine, 3–9 Fukuura, Kanazawa-ku, Yokohama, 236-0004 Japan; 5 Present Address: Department of Histopathology and Cytology, Japanese Red Cross Kyoto Daini Hospital, Kyoto, Japan

**Keywords:** Nephrotic syndrome, Nephron development, Laminin, Basement membrane, Extracellular matrix

## Abstract

**Background:**

Congenital nephrotic syndrome (CNS) is a rare disorder caused by various structural and developmental defects of glomeruli. It occurs typically as an isolated kidney disorder but associates sometimes with other systemic, extrarenal manifestations.

**Case Presentations:**

An infant presented with severe CNS, which progressed rapidly to renal failure at age of 3 months and death at 27 months. The clinical phenotypes and genetic causes were studied, including the renal pathology at autopsy.

Besides the CNS, the affected child had remarkable right-side predominant eye-ball hypoplasia with bilateral anterior chamber dysgenesis (microcoria). Brain MRI revealed grossly normal development in the cerebrum, cerebellum, and brain stem. Auditory brainstem responses were bilaterally blunted, suggesting a defective auditory system. At autopsy, both kidneys were mildly atrophied with persistent fetal lobulation. Microscopic examination showed a diffuse global sclerosis. However, despite of the smaller size of glomeruli, the nephron number remained similar to that of the age-matched control. Whole-exome sequencing revealed that the affected child was compound heterozygous for novel truncating *LAMB2* mutations: a 4-bp insertion (p.Gly1693Alafs*8) and a splicing donor-site substitution (c.1225 + 1G > A), presumably deleting the coiled-coil domains that form the laminin 5–2-1 heterotrimer complex.

**Conclusions:**

Our case represents a variation of Pierson syndrome that accompanies CNS with unilateral ocular hypoplasia. The average number but smaller glomeruli could reflect either mal-development or glomerulosclerosis. Heterogeneous clinical expression of *LAMB2* defects may associate with the difference in fetal β1 subtype compensation among affected tissues. Further study is necessary to evaluate incidence and features of auditory defect under LAMB2 deficiency.

**Electronic supplementary material:**

The online version of this article (doi:10.1186/s12882-017-0632-4) contains supplementary material, which is available to authorized users.

## Background

Congenital nephrotic syndrome (CNS) is a heterogeneous disorder that characteristically starts in utero or within the first 3 months of life. Recent cohort studies worldwide have demonstrated that CNS manifesting within the first year of life is mostly explained by mutations in any of the four genes *NPHS1*, *NPHS2*, *WT1*, and *LAMB2* [1–3]. CNS is usually a single-organ disorder that is restricted to the kidneys, but it can sometimes manifest other extrarenal developmental abnormalities, including in the neuronal, ocular, and skeletal systems. The genetic basis of the phenotypic variability underlying CNS is not completely understood.

Five to 10% of CNS cases are caused by genetic defects associated with the glomerular basement membrane (GBM) or extracellular matrix that covers the outermost glomerular capillary wall. The GBM is composed mainly of laminins and type-IV collagens [4–6]. These proteins play a key role in the migration, polarity, and differentiation of stem cells, and genetically caused aberrations in these molecules often lead to embryonic mortality or developmental defects in multiple organs. Laminins are generally composed of the α, β, and γ chains, which exist in five (α1–α5), four (β1–β4), and three (γ1–γ3) isoforms, respectively. These theoretically generate at least 60 different trimers, of which only 16 are biologically active in tissues. Combinations of the trimer subtypes and their relative abundance vary widely among the tissues and their developmental stages. Owing to the heteromeric nature of laminins and their spatiotemporal expression, remarkable phenotypic variations have been reported in human disorders caused by the laminin mutations [4–6].

One example of a genetic GBM defect is Pierson syndrome (PS, OMIM #609049), which is an autosomal recessive disorder characterized by the co-occurrence of CNS with various ocular anomalies [7]. The hallmark of the eye abnormalities is non-reactive narrowing of the pupils, so-called microcoria. PS is caused mostly by biallelic functional null variants of *LAMB2*, encoding a β2 subunit that forms a heterotrimeric laminin α5-β2-γ1 (LM-521) complex [8, 9]. The LM-521 complex is a major extracellular matrix that anchors the glomerular podocytes onto the mature GBM [4–6, 10]. The phenotypic spectrum of *LAMB2* mutations is much broader than previously thought [9]. Some milder missense *LAMB2* variants give rise to an isolated renal disorder [11]. In contrast, among the more than 80 *LAMB2* mutations reported so far, about 70% are truncating-type, non-functioning alleles. These biallelic null mutations lead to a complete loss of β2 chains in the majority of PS cases, and usually cause a wide variety of renal histology and ocular abnormalities [8, 12]. Most children with PS showed diffuse mesangial sclerosis (DMS), whereas others exhibit a minimal change or history of focal glomerulosclerosis [9]. Neurodevelopmental deficits, including hypotonia, intellectual disability, and speech delay, are reported in some patients with *LAMB2* mutations [13]. These observations suggest the complex nature of the phenotypic expression of mutations in this gene.

The aim of this study was to define as-yet uncharacterized features of *LAMB2*-based disorders, particularly focusing on renal histology as well as sensory organ involvement. Histologic examination of the autopsied renal tissues indicated that the nephrons had formed in a number similar to controls but appeared to be slightly smaller in size, suggesting that mal-development or glomerulosclerosis arose from the LAMB2 deficiency. The affected child showed hypoplastic and dysplastic eye structures as well as hearing disability. An awareness of these extrarenal features would help in the diagnosis of CNS due to laminin defects.

## Case Presentation

### Clinical findings

A 2-month-old Japanese girl was transferred from an affiliated clinic because of generalized edema and respiratory distress (Additional file 1). The infant was born at another hospital at 44 weeks gestation from a mother who was gravida 1, para 1. She was the first child of her parents, both of whom were healthy. There was no family history of kidney diseases. The pregnancy was uneventful, and serologic test for TORCH was negative. Her birth weight was 2.7 kg (−1.1 SD, 12.4 percentile), her body length was 47.4 cm (−1.2 SD, 10.0 percentile), and the placenta weighed 690 g. She also had ankyloglossia in the absence of lip and palate defects, which was treated by frenuloplasty at age of 2 months. There was no obvious facial dysmorphism. The girl lacked visual attention and was apparently blind from birth. The infant fed fairly well until 2 months of age, when she began to develop edema and oliguria.

Upon admission at age of 2 months, she had a height of 54 cm (−1.2 SD, 15 percentile) and weight of 5.3 kg (+0.5 SD, 55 percentile). An ophthalmologic examination revealed remarkable microphthalmia of the right eye. Magnetic resonance imaging (MRI) of the orbit revealed that the right eye ball was collapsed, with irregular thickening of the eye ball wall as well as lens dislocation (Additional file 2). In contrast with the remarkable ocular hypoplasia on the right side, the left eye ball was developed into grossly normal size. The axial length of the left eye globe was 21 mm, slightly greater than the normal range for her age (16.78 ± 0.51 mm) [14, 15]. Further ophthalmologic examination revealed bilateral anterior chamber dysgenesis, which was characterized by shallow chambers with missing (acorea, right) or small pupils (microcoria <2 mm in diameter). In the right eye, the cornea was clear under scotopic illumination. However, the pupil border was ill-defined because of the grayish, web-like remnant papillary membrane covering its anterior surface. In contrast, the left eye pupil was small but of regular round shape, and without any iris and corneal abnormalities. Brain MRI showed that the cerebrum, cerebellum, and brain stems were normal. The auditory brainstem responses were bilaterally absent (Additional file 3). On neurologic examination, her deep tendon reflexes and muscle tone were normal. Karyotyping was of a normal female karyotype. The newborn metabolic screen was negative.

The infant showed nephrotic syndrome with serum total protein of 1.8 g/dL, albumin of 0.6 g/dL, and spot urinary protein of 7.9 g/g creatinine (normal range, less than 0.7). Ultrasonography of the abdomen demonstrated that both kidneys were enlarged for her age: the axial length of the right kidney was 5.9 cm and that of the left kidney was 6.1 cm. She progressed to end-stage renal disease at age of 3 months and was started on peritoneal dialysis. At 12 months of age, both growth and developmental delays were noted: she had a height of 73.2 cm (45 percentile) and weight of 9.3 kg (70 percentile); motor milestones were severely delayed as the girl remained supine and did not acquire the ability to sit unaided. The patient eventually suffered from repeated infection and died from septic shock at 26 months of age, which probably resulted from the peritoneal dialysis-related peritonitis. An autopsy was performed.

### Autopsy findings

**Fig. 1:**
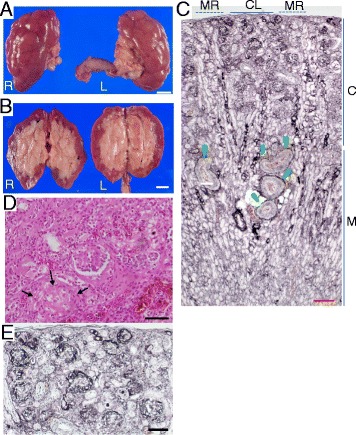
Macroscopic and microscopic findings of autopsied kidneys. **a, b,** *macroscopic view*. The both kidney were mildly atrophied with surface lobulation (Panel **a**, Bar = 1 cm). In a coronal slice of the kidney (Panel **b**, Bar = 1 cm), layer structural organization of cortex and medulla was preserved but the cortex was thinner in relative to medulla. **c, d, e,** *light microscopic view* Longitudinal section (Panel **c**) revealed the distribution and arrangement of glomeruli along the cortical labyrinth (CL) and the medullary rays (MR). There was diffuse interstitial fibrosis with tubular atrophy. Focal tubular dilatation was observed (*arrowhead*). PAM, 40× low power magnification, Bar = 200 μm. C:cortex; M:medulla. Higher magnification of cortical zone (Panel **d**, **e**) revealed that most glomeruli were globally sclerosed (*arrows*) with nearly total obliteration of capillary limen, suggestive of the diffuse loss in structural integrity of capillaries (D, HE 200×, E, PAM 100× magnification, Bar = 100 μm)

The left kidney weighed 35 g (axial length of 5.7 cm x width 3.0 cm) and the right kidney weighed 35 g (6.5 × 3.5 cm) (Fig. 1a), which were almost within average range for the patient’s age. Gross inspection of the cortical surface showed multiple indentations, suggesting persistent fetal lobulation. Cross-sections of the kidneys revealed that the distinction between cortex and medulla was preserved (Fig. 1b). There was remarkable thinning of the cortex, with a blunted protrusion of medullary papillary tips into the calyces. Light microscopic examination showed that the number of glomeruli was grossly normal (Fig. 1c). However, the glomeruli were globally sclerosed with nearly total obliteration of the capillary lumen (Fig. 1d, e). Morphometric analysis showed that the glomerular density in the cortex was slightly greater than that in the age-matched control (12.9 ± 0.8 vs. 8.8 ± 1.3, number per area in mm^2^) (Additional files 1 and 4). In contrast, the size of glomeruli in the affected child was slightly smaller (89.0 ± 1.6 μm) than that of the control (106.3 ± 3.3 μm), the values of which were close to the normal range previously reported (116–124 μm) [16]. Pronounced focal fibrosis and tubular atrophy were evident in the interstitium. The observations indicate that the glomeruli had developed almost normally in terms of the number but eventually progressed to diffuse and global sclerosis during the earlier periods of her life.

### Genetic analysis

**Fig. 2:**
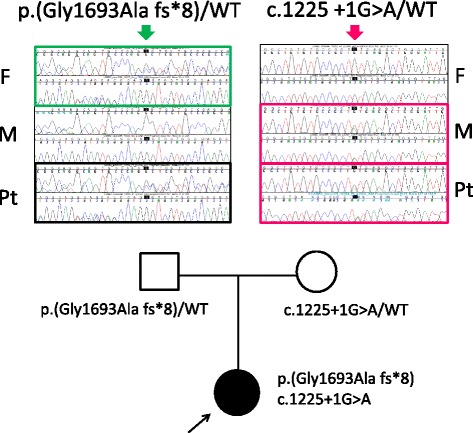
Mutational analysis of *LAMB2*. The patient was compound heterozygous for two mutations. One is paternal allele of splice-donor site mutation (c.1225 + 1G > A). Another is maternally transmitted, four base-pair insertion leading to early termination (c.5077_5078insCCAG;p.Gly1693Alafs*8). F: father; M: mother; Pt: patient; WT: wild-type. The nucleotide numbering is according to the reference sequence GenBank accession NM_002292.3 with the first nucleotide of the ATG start codon on position +1

**Fig. 3:**
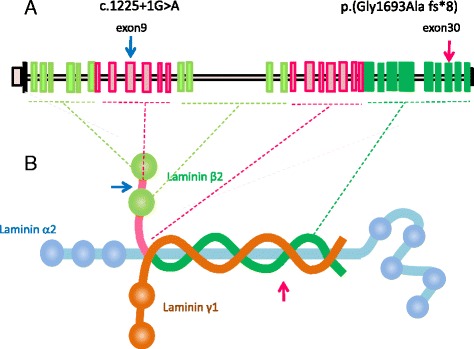
Locations of the *LAMB2* mutations and molecular structure of laminin-521. **a.** *exon-intron organization of LAMB2 gene*. The positions of two mutations found in the affected individual are indicated by *arrows.* **b**. st*ructure of laminin (LM)-521 and locations of mutations*. Laminin is a large cruciform heterotrimeric glycoprotein and comprises basal membranes. *LAMB2* encodes a Laminin subunit β2. It is a principal component of heterotrimer laminin α5β2γ1, which serves as a predominant extracellular matrix for mature GBM. The laminin α5β2γ1 is now referred to as LM-521 in the new nomenclature [10]. Trimers are stabilized via the coiled-coil long arm, while short arms are composed of globular and intervening LE domains and vary in size among the distinctive subunits. LE: laminin-type epidermal growth factor-like modules; G:globular domain The patient has biallelic *LAMB2* mutations: one truncates C-terminal 90 amino acids (*red arrow*), while another incorporates an earlier termination codon at N terminal one thirds position of β2 subunit through a splice error in a splice-donor +1 site of exon 9 (*blue arrow*). Both mutations likely induce a nonsense-mediated mRNA decay (NMD). The biallelic *LAMB2* mutations together impaired the assembly of the LM-521, thereby accounting for developmental abnormalities

Sequencing analysis demonstrated that the affected child was a compound heterozygote for *LAMB2* mutations: a 4-bp insertion (c.5077_5078insCCAG, exon 30) and a G to A substitution of the 1++1 splice donor site (c.1225 +1 G > A, exon 9) (Figs. 2 and 3, Additional file 1). Both mutations are novel. The former is paternally transmitted, whereas the latter is maternal. The c.5077_5078insCCAG insertion causes a frame shift, thereby truncating C-terminal 99 amino acids (p.Gly1693Alafs*8). The c.1225+1 G > A transition is predicted to activate a 60–90-bp downstream cryptic donor site, which subsequently incorporates an early termination codon. These variants were not found in 860 ethnic-matched healthy controls nor in human disease mutation database, indicating a novel and rare nucleotide change. The two mutations were likely subjected to nonsense-mediated mRNA decay, due to early transcriptional termination, and thus represent functional null alleles.

## Discussion

The present case represents a phenotypic variation of Pierson syndrome (MIM609049), which displayed a combination of developmental defects, including CNS, unilateral ocular hypoplasia, and deafness. Such multiorgan manifestations initially led us to suspect the diagnosis of PS. However, the lack of microcoria, a pathognomonic eye feature for PS, made it difficult to define the molecular causes until the final genetic determination of *LAMB2* mutations. The features of CNS with rapid progression to end-stage renal disease in our infant agreed with previous observations that severe PS in most patients is caused by homozygosity or compound heterozygosity for truncating *LAMB2* mutations [8, 9]. These mutations represent functional null alleles that are likely subjected to nonsense-mediated mRNA decay and therefore could disrupt a stable heterotrimeric laminin complex assembly in affected tissues (Additional file 5).

Upon histologic study of the autopsied renal tissues, we found that most of the glomerular capillaries were totally obliterated. The findings are consistent with DMS, as reported in the patients with functional null alleles of *LAMB2*. DMS is a histologic change resulting from a loss of glomerular capillary integrity and is frequently found in CNS patients with immature, underdeveloped glomeruli [17]. Our results, together with those of others, suggested that the laminin β2 chain is required for proper GBM filtration function after birth. Despite extensive studies in knockout mice [18–20], the role of laminin β2 in the development of human nephrons has remained elusive. Our morphometric analysis showed that the glomerular density of the patient was greater than that of the age-matched control. The results indicate that the absence of LAMB2, at least, does not reduce the number of nephrogenesis, a physiological process which actively occurs underneath the renal capsules. Even the greater glomerular density in the affected child may be due to the reduction of the cortical surface area associated with progressive nephron loss and/or defective migration of immature glomeruli from the nephrogenic zone to the deeper cortex. However, the glomerular size of the patient was significantly smaller than that of the age-matched control. This indicates that glomerulosclerosis progresses over the later embryonal stage or postnatal period. Our result is consistent with the observation that laminin β2 null mice (*lamb2*−/−) are born alive, and their GBM ultrastructure appears to be morphologically normal albeit creating some leaky nephrotic glomeruli [19, 20]. The absence of morphological abnormalities of GBM at birth is likely explained by the compensation of defective β2 by alternative fetus form of laminin β1 subunit. During glomerular maturation, the GBM undergoes a transition from LM-111 to LM-511 and finally to LM-521, which is the only isoform in the adult mature GBM [4, 6]. Laminin β1 could therefore partly compensate for the missing LM-521 [19, 20]. On the other hand, if the laminin α5 chain of LM-511 (α5-β1-γ1) and LM-521 is missing in mice, then renal agenesis is observed in 20% of the mutant embryos. Thus, the laminin α5 chain is indispensable for earlier nephrogenesis [6, 21]. Our data, together with studies by other investigators, suggest that laminin β2 deficiency does not reduce initial nephron number but will lead to glomerulosclerosis, which partly associates with a programed isoform switch from fetal β1 to adult β2 subtype towards the birth.

Our study highlighted a complexity of ocular developmental defects caused by *LAMB2* mutations. Various ocular anomalies previously reported in PS patients include megalocornea, iris hypoplasia, cataract, abnormal lens shape, posterior lenticonus, persistent fetal vasculature, retinal detachment, and glaucoma. This variability reflects the spatiotemporal expression of LAMB2 in the lens capsule and the Bowman layer, in the basal membranes surrounding the ciliary and iris muscles and the retina during fetal development [7, 8, 22, 23]. The unilateral microopthalmia found in our patient has been reported by other researchers [9, 12, 13]. A study of 34 eyes from patients genetically diagnosed with PS [12] demonstrated considerable interocular differences with regard to the severity in ocular phenotypes: about half of the eyes (18/34, 53%) had increased age-adjusted axial lengths, whereas one fifth (7/34, 21%) were microphthalmic. As seen in the present case, microopthalmia is invariably caused by truncating *LAMB2* mutations [12, 24]. Increased axial lengths and enlarged corneal diameter were seen in some cases. Extracellular matrix proteins, such as laminins, play various roles in ocular morphogenesis by regulating the developmental signals that govern specification, patterning, and differentiation of the optic cup [25]. The variability might be explained by developmental stage- or tissue-specific compensation mechanisms. Immature GBM bears β1, which is replaced by β2 as development proceeds [19]. Laminin β1 (or other laminin β isoforms) might be able to compensate for the missing β2 to some extent. The variable phenotypes could also arise in part from the deposition of multiple ectopic laminins (e.g., LM-511, LM-111, LM-211, etc.) [20] that do not normally exist in the GBM or the eye. The identification of possible modifiers of the phenotype caused by laminin β2 defects may also be important.

It is noteworthy to discuss some previously undescribed features in this case. Hearing disability has not previously been reported in PS. Histochemical study of the human cochlea has demonstrated that *LAMB2* is expressed in the basal membrane of the organ of Corti [26]. Our results suggest that physicians should pay more attention to the development of not only the visual system but also auditory function in children with CNS. Further study with more patients will be necessary to evaluate the clinical incidence and features of hearing loss under LAMB2 deficiency.

## Conclusions

In conclusion, our observations indicate that *LAMB2* deficiency did not reduce overall nephron number during the fetus stage, presumably due to the presence of alternate embryonic β1 isoform (LAMB1). However, as such compensation attenuates postnatally, the affected child showed severe nephrotic syndrome soon after birth and progressed into the end stage renal failure likely because of maldevelopment and/or sclerotic changes of the glomeruli. There is remarkable phenotypic variability in extra-renal features arising from among *LAMB2* mutations. Ocular anomalies include hypoplasia and dysplasia and are often more complex than originally reported. Hearing disability is likely previously unrecognized feature of LAMB2 deficiency. This heterogeneity may reflect a type of mutation, compensatory mechanisms among laminin subunits, and/or possible as-yet unknown modifier genes. Further studies will help toward understanding the phenotypic spectrum of *LAMB2*-based disorders.

## Additional files

Additional file 1: Patient and Methods. (PDF 375 kb)

Additional file 2: Clinical phenotype (1): MRI features of the orbit and brain. (PDF 369 kb)

Additional file 3: Clinical phenotype (2): Auditory brainstem responses. (PDF 419 kb)

Additional file 4: Clinical phenotype (3): Glomerular density and size in renal tissues. (PDF 441 kb)

Additional file 5: Scheme of disease mechanism: Basal attachment of the podocyte sole onto the glomerular basement membrane (GBM). (PDF 418 kb)

## Abbreviations

CNS: Congenital nephrotic syndrome
GBM: Glomerular basal membrane
*LAMB1*: Laminin isoform beta 1
*LAMB2*: Laminin isoform beta 2
