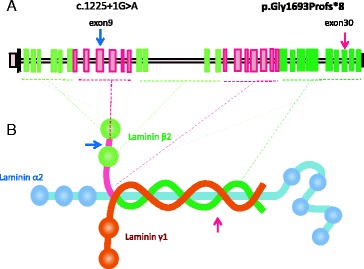# Erratum to: Nephron development and extrarenal features in a child with congenital nephrotic syndrome caused by null *LAMB2* mutations

**DOI:** 10.1186/s12882-017-0682-7

**Published:** 2017-08-17

**Authors:** Jiro Kin, Hiroyasu Tsukaguchi, Takahisa Kimata, Huan Thanh Nguyen, Yorika Nakano, Noriko Miyake, Naomichi Matsumoto, Kazunari Kaneko

**Affiliations:** 1 Department of Pediatrics, Kansai Medical University, 2-5-1 Shimachi, Hirakata, Osaka, 573–1010 Japan; 2 Second Department of Internal Medicine, Kansai Medical University, 2-5-1 Shinmachi Hirakata, Osaka, 573–1010 Japan; 3 Department of Pathology and Laboratory Medicine, Kansai Medical University, Osaka, Japan; 4 Department of Human Genetics, Yokohama City University Graduate School of Medicine, 3–9 Fukuura, Kanazawa-ku, Yokohama, 236–0004 Japan; 5 Present Address: Department of Histopathology and Cytology, Japanese Red Cross Kyoto Daini Hospital, Kyoto, Japan

## Erratum

In the original version of this article [1], published on 6 July 2017, the incorrect nomenclature “c.5077_5078insCCAG p.Gly1693Alafs*8” was used for the truncating mutation caused by the four-base pair change. The correct nomenclature for this mutation should be “c.5073_5076dup p. Gly1693Profs*8”, according to the sequence variant nomenclature of Human Genome Variant Society (HGVS: http://varnomen.hgvs.org/) as well as the Human Gene Mutation Database (HGMD: www.hgmd.org).

The correction of the nomenclature doesn’t impact the conclusion of the article. The extra 4 four-base-pair Indel, even in either original (mistakenly interpreted as the “CCAG” is inserted between the first [G] and second base [G] of Gly codon 1693 [G^∨^GT]: ^∨^ indicates the place where the mutation arises) or corrected nomenclature (“CCAG” is duplicated just one base-pair upstream to the first base [G] of Gly codon 1693 [∨GGT]), gives rise to the biologically same, frame-shift, truncated, “Null” LAMB2 protein.

The original version of this article [1] also contains a typographical error in the "Genetic analysis" and an additional error in the caption of "Fig. 2", where the words "paternal" and "maternally" are mixed up.

In this Erratum the original nomenclature has been listed (bold) and the corrected nomenclature has also been listed (bold). The same has been done for the typographical error and the additional error.

The typographical error has been corrected in the original publication. The nomenclature and the additional correction were not corrected in the original article.

### Original publication

1. **In the “Abstract”:**
  Whole-exome sequencing revealed that the affected child was compound heterozygous for novel truncating LAMB2 mutations: a 4-bp insertion (p.Gly1693Alafs*8) and a splicing donor-site substitution (c.1225 + 1G > A), presumably deleting the coiled-coil domains that form the laminin 5–2-1 heterotrimer complex.
2. **In the “Genetic analysis”:**
  Sequencing analysis demonstrated that the affected child was a compound heterozygote for LAMB2 mutations: a **4-bp insertion (c.5077_5078insCCAG**, exon 30) and a G to A substitution of the **1++1** splice donor site (c.1225 + 1 G > A, exon 9) (Figs. 2 and 3, Additional file 1). Both mutations are novel. The former is paternally transmitted, whereas the latter is maternal. The **c.5077_5078insCCAG insertion** causes a frame shift, thereby truncating C-terminal 99 amino acids (p.Gly1693Alafs*8).
3. **In “Fig. 2” and the caption:** 
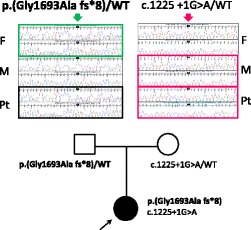

Figure 2 Mutational analysis of *LAMB2*. The patient was compound heterozygous for two mutations. One is **paternal** allele of splice-donor site mutation (c.1225 + 1G > A). Another is **maternally** transmitted, four base-pair insertion leading to early termination (c.5077_5078insCCAG;p.Gly1693Alafs*8). F: father; M: mother; Pt: patient; WT: wild-type. The nucleotide numbering is according to the reference sequence GenBank accession NM_002292.3 with the first nucleotide of the ATG start codon on position +1.

4. **In “Fig. 3”:** 
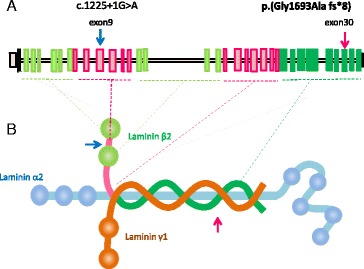

### Correct nomenclature/text

1. **In the “Abstract”:**
  Whole-exome sequencing revealed that the affected child was compound heterozygous for novel truncating LAMB2 mutations: a 4-bp duplication (Gly1693Profs*8) and a splicing donor-site substitution (c.1225 + 1G > A), presumably deleting the coiled-coil domains that form the laminin 5–2-1 heterotrimer complex.
2. **In the “Genetic analysis”:**
  Sequencing analysis demonstrated that the affected child was a compound heterozygote for LAMB2 mutations: a 4-bp duplication (c.5073_5076dup p.Gly1693Profs*8, exon 30) and a G to A substitution of the **+1** splice donor site (c.1225 + 1 G > A, exon 9) (Figs. 2 and 3, Additional file 1). Both mutations are novel. The former is paternally transmitted, whereas the latter is maternal. The **4-bp c.5073_5076 duplication** causes a frame shift, thereby truncating C-terminal 99 amino acids (p.Gly1693Profs*8).
3. **In “Fig. 2” and the caption:** 
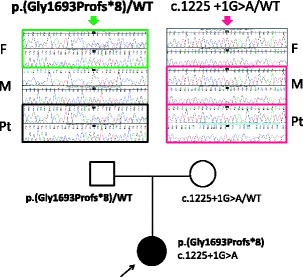

Figure 2 Mutational analysis of *LAMB2*. The patient was compound heterozygous for two mutations. One is **maternal** allele of splice-donor site mutation (c.1225 + 1G > A). Another is **paternally** transmitted, four base-pair insertion leading to early termination (c.5073_5076dup p.Gly1693Profs*8). F: father; M: mother; Pt: patient; WT: wild-type. The nucleotide numbering is according to the reference sequence GenBank accession NM_002292.3 with the first nucleotide of the ATG start codon on position +1.

4. **In “Fig. 3”:**