# Supplementary material for: Nephron development and extrarenal features in a child with congenital nephrotic syndrome caused by null LAMB2 mutations
Source: BMC Nephrol. 2017 Jul 6;18:220. doi: 10.1186/s12882-017-0632-4 (PMC5501564; doi:10.1186/s12882-017-0632-4)
Supplement: Supplementary file 1 — Patient and Methods. (PDF 375 kb) [file 12882_2017_632_MOESM1_ESM.pdf]

Additional file 1

Nephron Development and Extrarenal Features in a Child with Congenital Nephrotic Syndrome Caused by Null *LAMB2* Mutations.

Jiro kino; Hiroyasu Tsukaguchi; Takahisa Kimata; Huan Thanh Nguyen; Yorika Nakano; Noriko Miyake; Naomichi Matsumoto; Kazunari Kaneko

Patient and Methods

Study design and recruitment

We had taken care of this family in our hospital between 2012 and 2014. This case has not yet been reported elsewhere. After obtaining informed consent, clinical data and blood samples were obtained from individuals. The study protocol was approved by the Institutional Review Broad at the Kansai Medical University Hospital and Yokohama City University Faculty of Medicine.

Whole-exome sequencing

Genomic DNA was isolated from peripheral leukocytes using the QiaAmp DNA blood kit (Qiagen, Valencia, CA, USA). Whole-exome sequencing was performed for affected individuals (one patient from each family), as was reported previously [1]. In brief, 3 μg of genomic DNA was sheared using the Covaris S2 system (Covaris, Woburn, MA, USA), and genome partitioning was performed using the SureSelect Human All Exon V5 kit (Agilent Technology, Santa Clara, CA, USA) according to the manufacturer's instructions. The prepared samples were run on a HiSeq2000 instrument (Illumina, San Diego, CA, USA) with 101-bp paired-end reads and 7-bp index reads. The sequence reads were mapped to the hg19 reference human genome by Novoalign 3.00. Next, PCR duplication and variant calls were processed by Picard and Genome Analysis Toolkits. Based on the autosomal recessive model, homozygous or compound heterozygous variants were determined in each patient.

Variants with a minor allele frequency >0.005 in the Exome Variant Server, Exome Aggregation Consortium Browser, Human Genetic Variation Database, and in-house exome data (n = 575) were excluded from the study [2]. Genes harboring recessive variants detected commonly in

two or more probands were selected. Candidate recessive variants were checked in each family by Sanger sequencing to confirm the co-segregation of variants with the disease. Mutation data were described using the nomenclature of the Human Genome Variation Society recommendations; nucleotide and residue positions are given in compliance with the reference sequences published in the human GRCh38 assembly. The dbSNP (version 135) and 1000 Genomes (1,094 subjects of various ethnicities; May 2011 data release) public databases were also used.

Sanger sequencing

PCR primers flanking the entire coding region (exons 1–33) and flanking intron sequences of gene *LAMB2* were used as previously described [3]. The resulting PCR products were sequenced using the BigDye Terminator 3.1 reagents on an ABI 3500 analyzer. The *LAMB2* reference sequence used was NM_002292.3, in which the A of the ATG translation initiation codon was nucleotide 1. Parental genotypes were screened to assess whether the variant was *de novo* or inherited.

Morphometry of glomeruli

Sections (3-μm) of paraffin-embedded renal cortex specimen from the affected child (formalin-fixed autopsy samples) and from 5 controls (needle biopsies from those who presented minimal change in glomerular histology, at age 2–5 years) were used. The glomeruli were photographed at low magnification (40x, 100x), using the Olympus IX 73" microscope with the "CellSense" software (version 1.6; Olympus, Japan). The glomerulus was defined as the minimal convex polygon comprising the capillary tufts [4]. The size was evaluated by measuring the maximum linear distance across the glomerular tufts (diameter in mm). If an ellipsoid is randomly sliced at by a plane (as is the case when the kidney is sectioned, given the glomerulus is approximately an ellipsoid), the areas of the resulting ellipses will not precisely follow a symmetrical Gaussian distribution; there will be a relative excess of smaller areas, causing the distribution to be skewed to the left. However, the degree of non-normality is not sufficiently great to invalidate the two-tailed Student *t* test for the difference of the means of two groups of independent observations with unequal variances [5]. For glomerular density, the number of

glomeruli was manually counted from the cortical area just underneath the capsule. The adjacent consecutive fields (n = 2–4) representing an area of 1.0 mm^2 were chosen in each count [6].

References

1） Miyake N, Tsukaguchi H, Koshimizu E, Shono A, Matsunaga S, Shiina M, Mimura Y,Imamura S, Hirose T, Okudela K, Nozu K, Akioka Y, Hattori M, Yoshikawa N, Kitamura A, Cheong HI, Kagami S, Yamashita M, Fujita A, Miyatake S, Tsurusaki Y, Nakashima M, Saitsu H, Ohashi K, Imamoto N, Ryo A, Ogata K, Iijima K, Matsumoto N. Biallelic Mutations in Nuclear Pore Complex Subunit NUP107 Cause Early-Childhood-Onset Steroid-Resistant Nephrotic Syndrome. Am J Hum Genet. 2015; 97(4):555-566.

2） Higasa K, Miyake N, Yoshimura J, Okamura K, Niihori T, Saitsu H, Doi K, Shimizu M, Nakabayashi K, Aoki Y, Tsurusaki Y, Morishita S, Kawaguchi T, Migita O, Nakayama K, Nakashima M, Mitsui J, Narahara M, Hayashi K, Funayama R, Yamaguchi D, Ishiura H, Ko WY, Hata K, Nagashima T, Yamada R, Matsubara Y, Umezawa A, Tsuji S, Matsumoto N, Matsuda F. Human genetic variation database, a reference database of genetic variations in the Japanese population. J Hum Genet. 2016; 61(6):547-553.

3） Zenker M, Aigner T, Wendler O, Tralau T, Muntefering H, Fenski R, Pitz S, Schumacher V, Royer-Pokora B, Wuhl E, Cochat P, Bouvier R, Kraus C, Mark K, Madlon H, Dotsch J, Rascher W, Maruniak-Chudek I, Lennert T, Neumann LM, Reis A. Human laminin beta2 deficiency causes congenital nephrosis with mesangial sclerosis and distinct eye abnormalities. Hum Mol Genet 2004; 13:2625-2632

4） Lane PH, Steffes MW, Mauer SM. Estimation of glomerular volume: a comparison of four methods. Kidney Int. 1992;41(4):1085-1089.

5） Cohen AH. Massive Obesity and the Kidney. Am J Pathol 1975; 81:117-130.

6） Mañalich R, Reyes L, Herrera M, Melendi C, Fundora I. Relationship between weight at birth and the number and size of renal glomeruli in humans: a histomorphometric study. Kidney Int. 2000; 58(2):770-773.
