# Supplementary material for: Nephron development and extrarenal features in a child with congenital nephrotic syndrome caused by null LAMB2 mutations
Source: BMC Nephrol. 2017 Jul 6;18:220. doi: 10.1186/s12882-017-0632-4 (PMC5501564; doi:10.1186/s12882-017-0632-4)
Supplement: Supplementary file 2 — Clinical phenotype (1): MRI features of the orbit and brain. (PDF 369 kb) [file 12882_2017_632_MOESM2_ESM.pdf]

Additional file 2: Clinical phenotype (1)

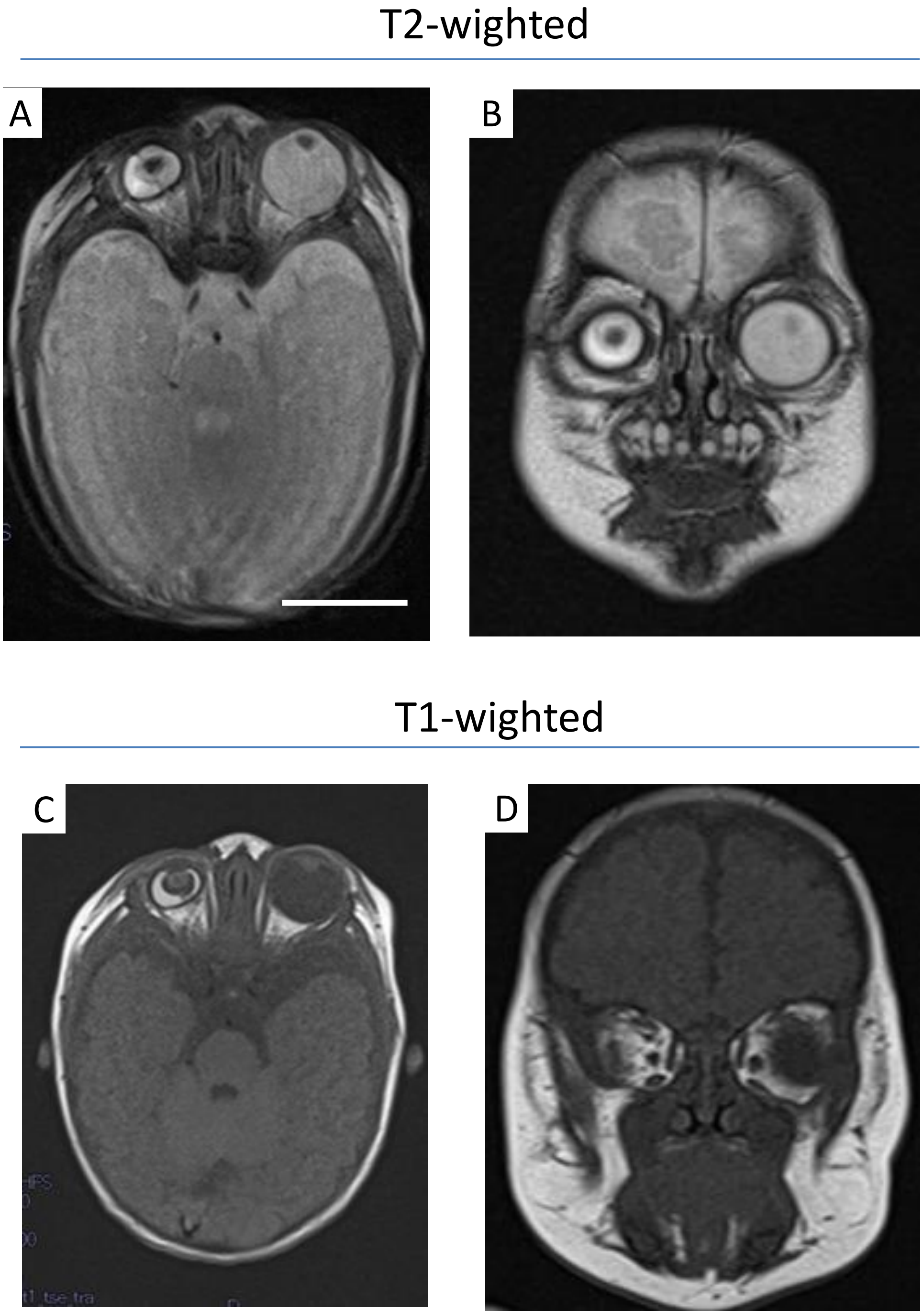

Additional file 2 MRI features of the orbita and brain
T2-weighted image (**A** axial, **B** coronal) and T1-weighted image (**C** axial, **D** coronal) are shown. The right eye ball was remarkably hypoplastic with lens dislocation and thickening of the eye globe wall. The size and organization of left eye appeared grossly normal. The lens was positioned to the anterior chambers but deviated toward upward (*arrowhead*) Optic nerves appeared normal bilaterally (*arrows*). Bar indicate 3cm.
