# Supplementary material for: Nephron development and extrarenal features in a child with congenital nephrotic syndrome caused by null LAMB2 mutations
Source: BMC Nephrol. 2017 Jul 6;18:220. doi: 10.1186/s12882-017-0632-4 (PMC5501564; doi:10.1186/s12882-017-0632-4)
Supplement: Supplementary file 3 — Clinical phenotype (2): Auditory brainstem responses. (PDF 419 kb) [file 12882_2017_632_MOESM3_ESM.pdf]

Additional file 3: Clinical Phenotype (2)

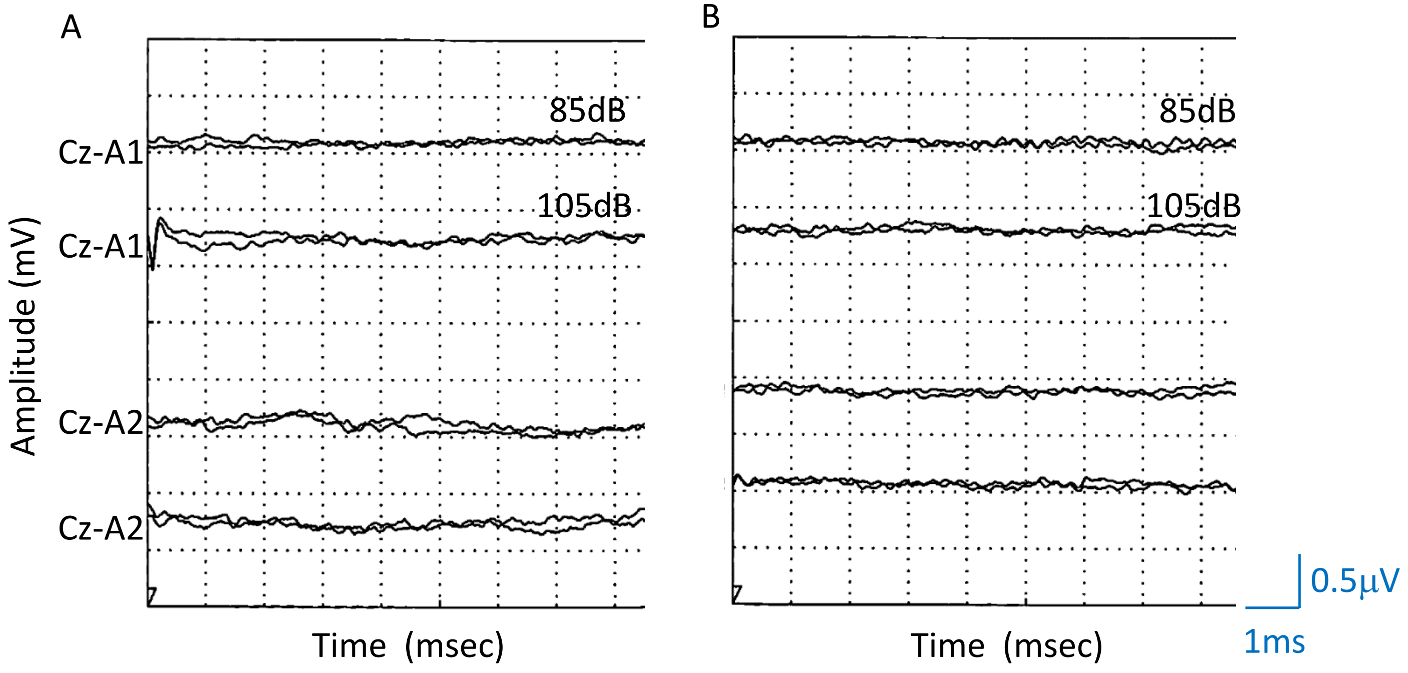

Additional file 3 Auditory brainstem responses

Findings at the age of 11 month old for the left (panel A) and right (panel B) ears are shown. Short latency evoked potentials are measured in response to the auditory click stimuli of 10 Hz with intensity range of up to 85 and 105 dB(Nihon Kohden, MB-2200). The filter was set at low cut 100 Hz and high cut at 3 kHz electrical interference from the surrounding electrical appliances. Tracing of amplitude and timing of electrical waves were recorded, where the electrodes were placed on parietal (Cz) and mastoid process points (A1: *left*, A2: *right*). No responses were evoked in both ears.
