# Supplementary material for: Nephron development and extrarenal features in a child with congenital nephrotic syndrome caused by null LAMB2 mutations
Source: BMC Nephrol. 2017 Jul 6;18:220. doi: 10.1186/s12882-017-0632-4 (PMC5501564; doi:10.1186/s12882-017-0632-4)
Supplement: Supplementary file 4 — Clinical phenotype (3): Glomerular density and size in renal tissues. (PDF 441 kb) [file 12882_2017_632_MOESM4_ESM.pdf]

Additional File 4 Clinical Phenotype (3)

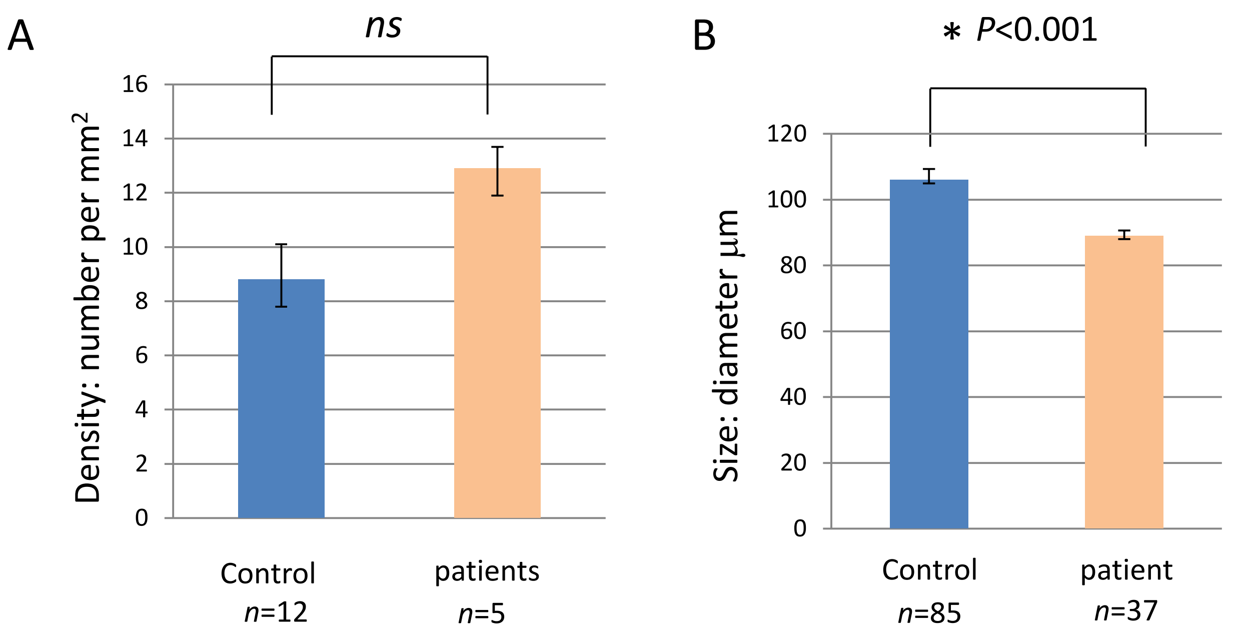

Additional file 4 Glomerular density and size in renal tissues
Glomerular density and diameter were compared between the affected individual and age-matched controls. ***A.,* glomerular density** within distinctive areas of cortex underneath renal capsule was counted in affected child (*n*=5, autopsied specimen) and control (*n*=12, needle biopsied). *ns*: not significant. ***B.,* glomerular size** average diameter of glomeruli in subcortical area (*n*=37, autopsied specimens) was measured for affected child in comparison with controls (*n*=85, needle biopsied) from 5 different individuals of age-matched children. Data are shown in mean± SE.
