# Supplementary material for: Nephron development and extrarenal features in a child with congenital nephrotic syndrome caused by null LAMB2 mutations
Source: BMC Nephrol. 2017 Jul 6;18:220. doi: 10.1186/s12882-017-0632-4 (PMC5501564; doi:10.1186/s12882-017-0632-4)
Supplement: Supplementary file 5 — Scheme of disease mechanism: Basal attachment of the podocyte sole onto the glomerular basement membrane (GBM). (PDF 418 kb) [file 12882_2017_632_MOESM5_ESM.pdf]

Additional file 5 Scheme of disease mechanism

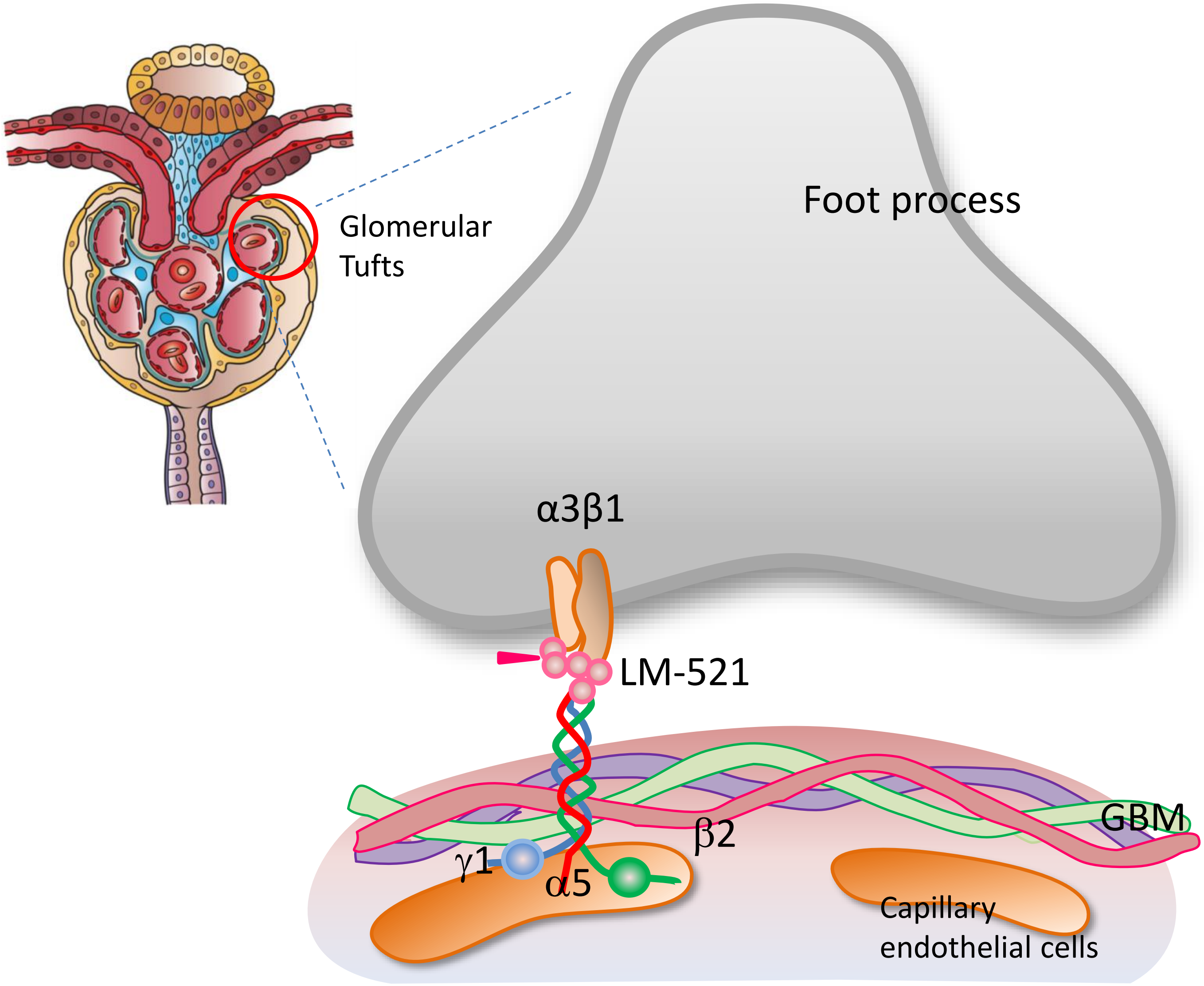

Additional file 5 Basal attachment of the podocyte sole onto the glomerular basement membrane(GBM).

The globular (G, *red arrowhead*) domain of an unprocessed laminin α5 chain (in LM-521) binds integrin receptor (IR) α3β1 subunit. A loss-of-function of the β2 subunit (*green*) likely impairs the anchoring of the cell to GBM, thereby leading to the defective polarity and differentiation of the podocytes. The cartoon original ly created ted by Domogatskaya A., et al (*Annu. Rev. Cell Dev. Biol*. 2012. 28:523–53) was modified with permission.
